# A comparison of flexural strengths of polymer (SBR and PVA) modified, roller compacted concrete

**DOI:** 10.1016/j.dib.2015.06.023

**Published:** 2015-07-08

**Authors:** John N. Karadelis, Yougui Lin

**Affiliations:** Department of Civil Engineering Architecture and Building, Faculty of Engineering and Computing, Coventry University, Coventry CV1 5FB, West Midlands, UK

**Keywords:** Flexural-performance, Polyvinyl-Alcohol, Styrene-Butadiene-Rubber

## Abstract

This brief article aims to reveal the flexural performance, including the equivalent flexural strength of PVA (Polyvinyl Alcohol) modified concrete by comparing it primarily with that of SBR (Styrene Butadiene Rubber) concrete. This data article is directly related to Karadelis and Lin [6].

**Table:** Specifications table

Subject area	Engineering and civil engineering
More specific subject area	Highways and transportation engineering
Type of data	Text file, tables, graphs and figures
How data was acquired	Mainly by a series of experimental (laboratory) investigations
Data format	All data were used as collected (raw). No statistical or any other treatment has taken place prior to analysis. However, as it is nearly always the case with engineering data type, they were carefully analysed and discussed and some useful conclusions were drawn.
Experimental factors	Two types of polymers, SBR (Styrene Butadiene Rubber) and PVA (Polyvinyl Alcohol) and two types of steel fibre, 35 mm and 50 mm long were used in mortar and concrete mixes. Concrete specimens (beams of 80×100×500) were fabricated in steel moulds using a purposely made vibrating compactor.
	SBR beams were cured in water for five days. PVA beams were cured in water for seven days, followed by air curing to testing. The ages of specimens prior to testing were 28–40 days.
Experimental features	Efforts were directed towards the representative test methods for steel fibre reinforced concrete currently available, the ASTM and BS were followed, where possible. More details (and the exceptions) are given below. Strengths were measured after adopting a 3PB (three point bending) test for reasons explained and justified later in the text (Fig. 2(c) and (d)).
Data source location	Department of Civil Engineering Architecture and Building, Faculty of Engineering and Computing, Coventry University, Coventry, W. Midlands, CV1 5FB, UK
Data accessibility	Data with this article

Value of the data

• These data is of significant value because, to the awareness of the authors, there are no previous records of the mechanical performance of steel fibres added in polymer modified concrete mix, made specifically for roller compaction.
• As the flexural performance of PVA modified concrete has not been fully investigated up till now, this data in brief article will serve as a ‘benchmark’ to the research community. It is hoped that it warrants motivation and follow up by other investigators for further research.
• The efficiency of fibres in the roller compacted, polymer modified concrete, that is, their contribution in resisting the opening and propagation of a crack and the ensuing development of the fibre bridging law should be of significant value to all those dealing with other than conventionally reinforced concrete.

## 1 Data, experimental design, materials and methods

A new material suitable for the structural repair of concrete pavements has been developed at Coventry University exhibiting high flexural, shear and bond strengths and high resistance to reflection cracking; at the same time demonstrating unique “*placeability”* and “*compactability”* properties.

There are many different products of the PVA family. A particular PVA was used by Hughes and Lubis [2] to modify cement mortar (MCM). High flexural strength and high bond strength with the steel reinforcement were achieved using a small roller compactor in the laboratory. Details about the PVA product, such as its name and manufacturer, are not available in their paper.

In this study, two PVA products, GH-17S and NH-18S, supplied by NIPPON GOHSEI [3] of Japan, were experimentally investigated. For more details please see reference Karadelis and Lin [6].

### 1.1 Data

**Fig. 1:**
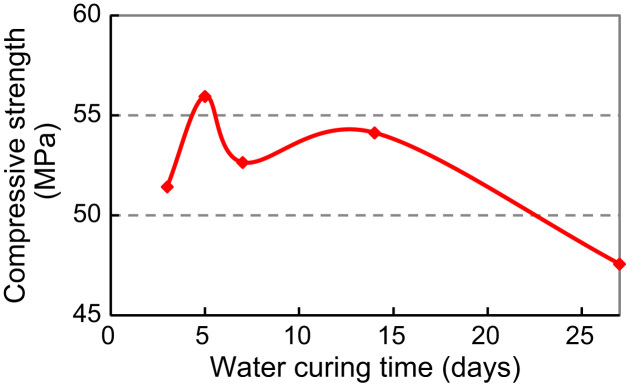
Relationship of cube strength with water curing times: SBR–MCM

SBR content is defined as the ratio of SBR solid to cement by weight, while the PVA content is the ratio of PVA to cement by weight. The water in the column of ‘mix proportion’ listed in Table 1 is the added water, that is, not including the water already contained in SBR. All cubes tested were of 50×50×50 mm^3^. They were fabricated with the help of a ‘hammer’ due to their glue-like behaviour (Fig. 1, Table 2).

**Fig. 2:**
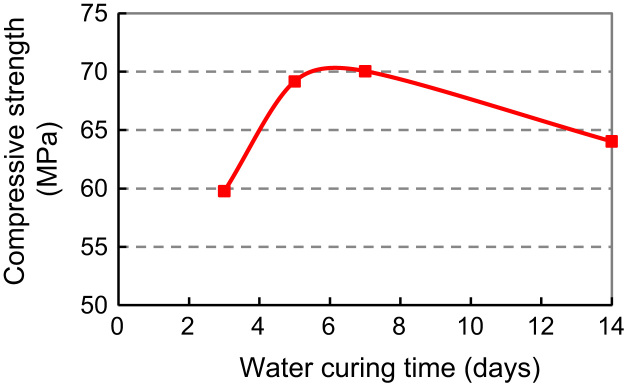
Relationship of cube strength with water curing times: PVA–MCM

It can be seen that the most favourable conditions for the two types are as follows:

For SBR–MCM (Modified Cement Mortar), 5-day water curing followed by 22-day air curing and for PVA–MCM, 7-day water curing followed by 20-day air curing. Thus, the above two curing procedures for SBR and PVA modified cement mortar and concrete were implemented in the study to follow (Fig. 2).

The optimum degree of SBR modification is usually achieved between 7.5% and 20% dry polymer solids by mass of cement in the mixture [1]. However, the use of SBR in excess is not economical, can cause excessive air entrainment and lead to strength loss. Furthermore, laboratory work conducted by the authors indicated that the addition of PVA resulted in poor workability of the mix; the higher the PVA dosage the stickier the mix became. Even a low PVA dosage, such as 1% to 3%, influenced distinctly the workability of the mix.

**Fig. 3:**
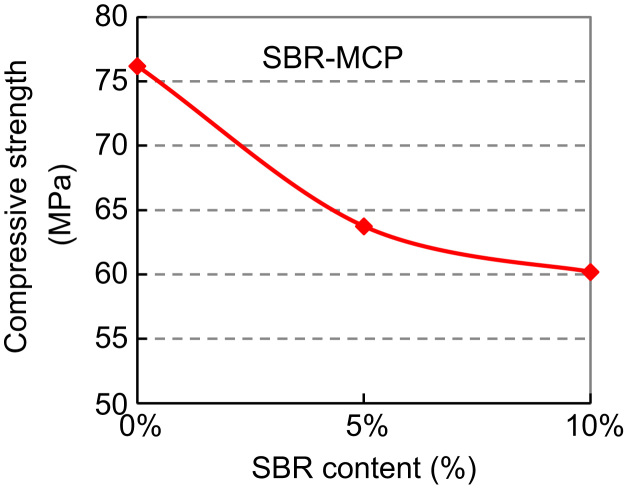
Compressive (cube) strength versus SBR content: SBR–MCP.

**Fig. 4:**
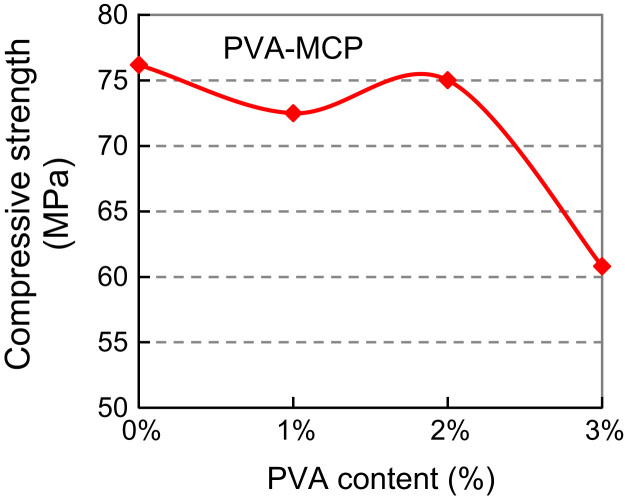
Compressive (cube) strength versus PVA content: PVA–MCP.

**Fig. 5:**
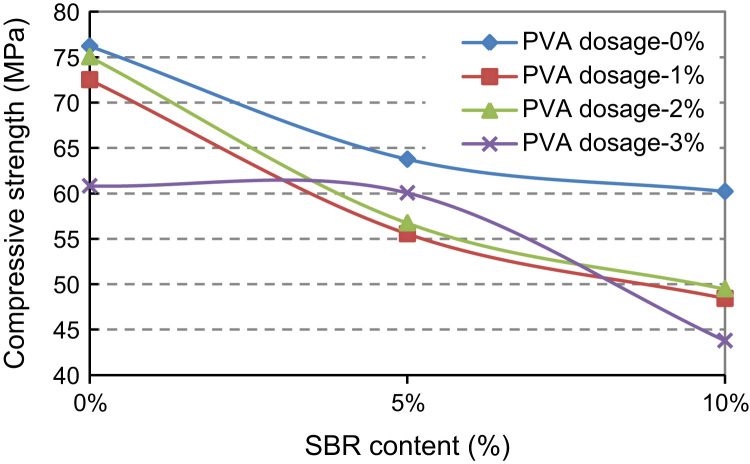
Compressive strength versus SBR content: SBR+PVA–MCP (hybrid) polymer.

Based on the above, SBR modified cement paste (SBR–MCP) with dosage of 5% and 10%, and PVA modified cement paste (PVA–MCP) with dosage of 1% and 2% and 3% were tested to explore the relationship between cube strength and polymer dosage and provide a contrast framework of the two. Cube specimens of 50 mm edge were prepared. Mix proportions are shown in Tables 3–5. The water-cement ratio was 0.230 for all mixes. Specimen preparation and test procedures were typical. The curing procedure for SBR–MCP was 5-day water and 22-day air curing; that for PVA–MCP was 7-day water and 20-day air curing. The 28-day cube strengths are listed in Tables 3–5 and plotted in Figs. 3–5.

The addition of SBR reduced distinctly the compressive strength of SBR–MCP mix. This is mainly attributed to air entrainment. Thus, it seems that the SBR dosage of 10% is optimum for obtaining high bond strength.

PVA dosage of 2% appeared to achieve the highest compressive strength of PVA–MCP mix. In the meantime the mix with the PVA dosage of 3% became very sticky, unworkable, and difficult for placement and formation. Thus, the PVA dosage of 2% was adopted as optimum after considering the criteria for strength and mix workability.

SBR+PVA hybrid modified cement paste was influenced by both polymers. The former affected mainly the compressive strength and the latter the workability. Therefore, based on the analyses above, 10% SBR and 2% PVA were considered to be optimum dosages for strength and workability. Further studies/results of concrete mixes with the optimal amount of polymers are presented below. The SBR+PVA hybrid polymer (10% SBR+2% PVA) was also used to study and enhance the bond strength.

The authors [7] conducted direct shear tests with composite cylinders, and performed splitting tests with composite blocks, to measure the interface bond strength between

a) SBRPMC–OPCC (Styrene Butadiene Rubber Polymer Modified Concrete onto Ordinary Portland Cement Concrete);
b) (SBR+PVA)PMC–OPCC (Styrene Butadiene Rubber plus Polyvinyl Alcohol Polymer Modified Concrete onto Ordinary Portland Cement Concrete); and
c) OPCC onto OPCC composite specimens.

The OPCC bases were at least 14 days old prior to placing the PMC parts. The polymer modified concrete (PMC) layers were placed using a specially designed vibrating compactor. The results were published in the reference by Lin et al. [7]. The mix containing the hybrid polymers, i.e. 10% SBR and 2% PVA developed significantly higher bond strengths than the rest.

Briefly, the direct shear bond strength and splitting tensile bond strength were as follows:

For the hybrid polymer, (a), they were 6.07 MPa and 2.56 MPa for 28-day old and 6.81 MPa and 3.43 MPa for 42-day old specimens.

For the SBRPMC–OPCC, (b), strengths were 5.47 MPa and 2.2 1MPa for 28-day old specimens.

Finally, for OPCC–OPCC, (c), strengths were 4.09 MPa and 2.17 MPa respectively.

Valid test methods for steel fiber reinforced concrete currently available are: the British Standard (BS) method [4], using a three-point bending (3PB) test on a notched beam, and crack mouth opening displacement (CMOD) as control. The ASTM method [5] that tests an un-notched beam under four-point bending (4PB) conditions, and mid-span deflection control. The ASTM method [5] was first tried, to evaluate the flexural strength of the mixes.

**Fig. 6:**
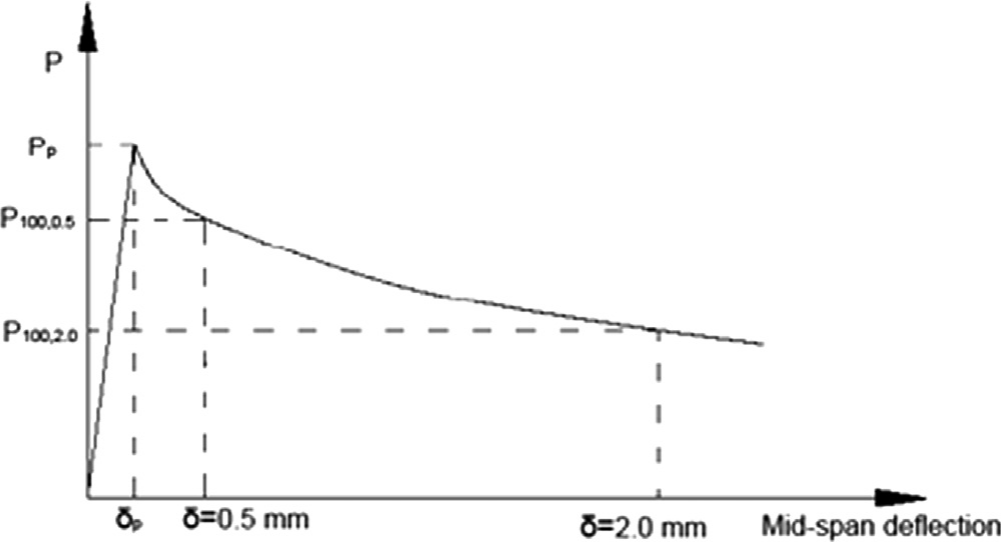
Chart used for the calculation of flexural strength [5].

**Fig. 7:**
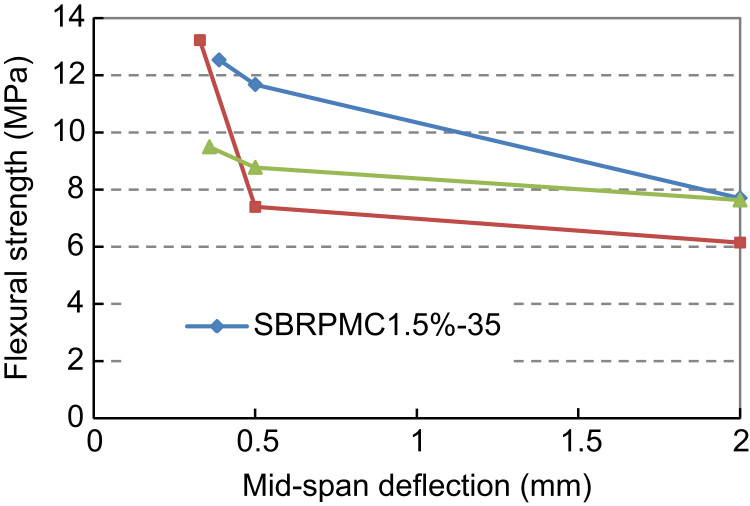
Flexural Strength of beams of three different mixes under 4PB tests.

Although the rate of increase of net deflection was within the range recommended by ASTM (2006), tests proved that the load increments were unsuitable, resulting in abrupt failure of three beams. Hence, the complete load mid-span deflection history for these beams is, regrettably, not available. The maximum flexural strength (*f*_*p*_) and residual flexural strengths (*f*_*R*,0.5_ and *f*_*R*,2_) were calculated by substituting data collected from the tests into Eq. (1), in accordance with ASTM C 1609/C 1609M-06 [5]. Fig. 7 displays the results.

(1) $$f_{j}=\frac{300P_{j}}{Bh^{2}}$$

where, *j*=*P* or *j*=*R*,0.5 or *j*=*R*,2; and *P*_*p*_, *P*_*p*,0.5_, *P*_*p*,2_, *f*_*p*_, *f*_*R*,0.5_ and *f*_*R*,2_ can be extracted from Fig. 6; *B* and *h* are the width and height of the beam, respectively.

The code name of the specimens was chosen as follows: SBRPMC 1.5%-35 is Styrene Butadiene Rubber Polymer Modified Concrete, containing 1.5% Steel fibers (by volume) of 35 mm length. Laboratory work showed that all SBRPMC 1.5%-35 and PVAPMC 1.5%-35 beams failed with multiple cracking under the four-point bending test. However, for concrete used as overlay on worn concrete pavements, a single reflective crack will initiate from the location of underlying existing cracks of worn pavements. The four-point bending test was deemed to be not suitable for testing overlay concrete, where the overlays would fracture at the location of underlying existing cracks. Thus, hereafter, the three-point bending test (3PB) was adopted to ensure a single crack development in the beam in failure.

### 1.2 Maximum and residual flexural strengths

**Fig. 8:**
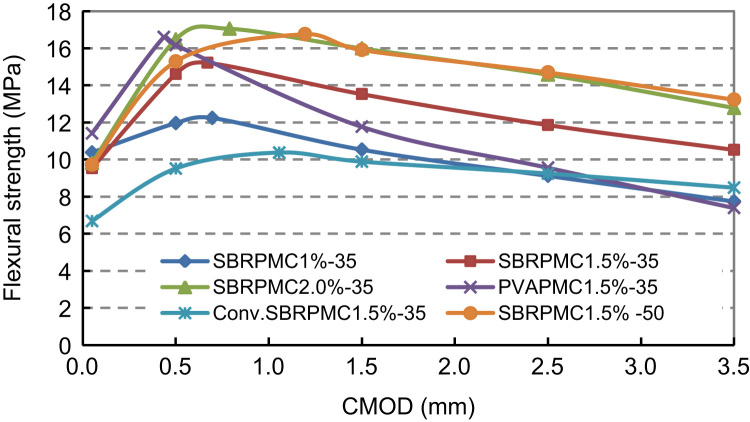
Flexural strengths of six PMC notched beams under 3PB test.

According to BS (BS EN 14651:2005+A1:2007, 2007), maximum flexural strength (*f*_*p*_), limit of proportionality $\left(f_{ct,L}^{f}\right)$, and residual flexural strengths $\left(f_{R,0.5},\;f_{R,1.5},\;f_{R,2.5}\;\mathrm{and}\;f_{R,3.5}\right)$, corresponding to CMOD_1_=0.5 mm, CMOD_2_=1.5 mm, CMOD_3_=2.5 mm and CMOD_4_=3.5 mm are evaluated to assess the flexural strengths using Eqs. (2)–(4) from the same standard. The number of specimens, the average flexural strengths and their standard deviation (STDEV) for each group are listed in Table 6, and the variation of flexural strength with CMOD for different mixes is plotted in Fig. 8.

(2) $$f_{\text{ct},L}^{f}=\frac{3SP_{L}}{2Bh_{sp}^{2}}$$

(3) $$f_{R,j}=\frac{3SP_{j}}{2Bh_{\mathrm{sp}}^{2}}$$

(4) $$f_{P}=\frac{3SP_{P}}{2Bh_{sp}^{2}}$$

with

$$h_{sp}=(h-a_{0})=(\text{hight}\;\text{of}\;\text{beam}-\text{depth}\;\text{of}\;\text{notch})$$

Although mix PVAPMC 1.5%-35 is not the best from the toughness and workability point of view, it exhibited high bond strength with the old concrete and therefore that influenced its choice. The mix proportions are listed in Table 1 [6]. Mix SBRPMC 1.5%-35 can be regarded as the optimal mix considering both, strength and workability. In particular, its flexural strength at early ages was very high, and hence it was deemed to be a suitable mix for worn concrete pavement rehabilitation.

## Figures and Tables

**Table 1:** Mix proportion of SBR–MCM and PVA–MCM.

Mix ID of PMM	SBR or PVA content	Mix parameter	Mix proportion
SBR–MCM	10%	SBR×46%=*C*×10% (SBR×54%+*W*)/*C*=0.206	*C*:S:SBR:*W*=1:1.26:0.217:0.0889
PVA–MCM	2%	*W*/*C*=0.261, PVA/*C*=2% Sup./*C*=1.5%	*C*:Sup.:Sand:PVA:*W*=1:0.015:1.26:0.02:0.261

**Table 2:** Cube strengths of SBR–MCM and PVA–MCM mixes from Table 1 at various water curing times.

Water curing time (days)		3	5	7	14	27
SBR–MCM cube strength	Average	51.43	55.95	52.65	54.13	47.57
(MPa)	STDEV	5.32	1.35	1.7	4.96	1.19
PVA–MCM cube strength	Average	59.77	69.17	70.04	64.03	
(MPa)	STDEV	10.85	6.30	1.42	7.49	

**Table 3:** Mix proportion and cube strength of SBR–MCP with different SBR content.

SBR content (%)	Mix parameter	Mix proportion	Cube strength (MPa)	Description
0	*W*/*C*=0.230	*C*:*W*=1:0.230	76.19	5-day water curing
5	SBR×46%=*C*×5% (SBR×54%+*W*)/*C*=0.23	*C*:SBR:*W*=1:0.109:0.171	63.74	22-day air curing
10	SBR×46%=*C*×10% (SBR×54%+*W*)/*C*=0.23	*C*:SBR:*W*=1:0.217:0.117	60.19	Sticky behaviour

(*C*, SBR, *W*)=(Cement, SBR and water) in mass.

**Table 4:** Mix proportion and cube strength of PVA–MCP with different PVA content.

PVA content (%)	Mix parameter	Mix proportion	Cube strength (MPa)	Description
0	*W*/*C*=0.230	*C*:*W*=1:0.230	76.19	7-day water curing 20-day air curing
1	PVA=*C*×1% *W*/*C*=0.230	*C*:PVA:*W*=1:0.01:0.230	72.5	
				Sticky behaviour
2	PVA=*C*×2% *W*/*C*=0.230	*C*:PVA:*W*=1:0.02:0.230	75.02	
3	PVA=*C*×3% *W*/*C*=0.230	*C*:PVA:*W*=1:0.03:0.230	60.81	

(*C*, PVA, *W*)=(Cement, PVA and water) in mass.

**Table 5:** Mix proportion and cube strengths of SBR & PVA hybrid polymer modified cement paste.

SBR cont	PVA conte. (%)	Mix parameter	Mix proportion	Compressive strength (MPa)
0%	0	*W*/*C*=0.230	*C*:*W*=1:0.230	76.19
	1	PVA=*C*×1% *W*/*C*=0.230	*C*:PVA:*W*=1:0.01:0.230	72.5
	2	PVA=*C*×2% *W*/*C*=0.230	*C*:PVA:W=1:0.02:0.230	75.02
	3	PVA=*C*×3% *W*/*C*=0.230	*C*:PVA:*W*=1:0.03:0.230	60.81
5%	0	SBR×46%=*C*×5% (SBR×54%+*W*)/*C*=0.230	*C*:SBR:*W*=1:0.109:0.171	63.74
	1	SBR×46%=*C*×5% PVA=*C*×1% (SBR×54%+*W*)/*C*=0.230	*C*:PVA:SBR:*W*=1:0.01:0.109:0.171	55.53
	2	SBR×46%=5%×*C* PVA=*C*×2% (SBR×54%+*W*)/*C*=0.230	*C*:PVA:SBR:*W*=1:0.02:0.109:0.171	56.73
	3	SBR×46%=5%×*C* PVA=*C*×3% (SBR×54%+*W*)/*C*=0.230	*C*:PVA:SBR:*W*=1:0.03:0.109:0.171	60.04
10%	0	SBR×46%=*C*×10% (SBR×54%+*W*)/*C*=0.230	*C*:SBR:*W*=1:0.217:0.117	60.19
	1	SBR×46%=*C*×10% PVA=*C*×1% (SBR×54%+*W*)/*C*=0.230	*C*:PVA:SBR:*W*=1:0.01:0.217:0.083	48.41
	2	SBR×46%=*C*×10% PVA=*C*×2% (SBR×54%+*W*)/*C*=0.230	*C*:PVA:SBR:*W*=1:0.02:0.217:0.083	49.47
	3	SBR×46%=*C*×10% PVA=*C*×3% (SBR×54%+*W*)/*C*=0.230	*C*:PVA:SBR:*W*=1:0.03:0.217:0.0833	43.77

**Table 6:** Flexural strengths of various mixes under 3PB test (all beams tested were of the same height of 100 mm and the same ligament depth of 80 mm)

ID of specimens	No. of beams	Statistics	Limit of prop		Maxim strength		Residual flexural strength			
			f_0.05_	CMOD	*f*_*P*_	CMOD	*f*_*R*,0.5_	*f*_*R*,1.5_	*f*_*R*,2.5_	*f*_*R*,3.5_
			(MPa)	(mm)	(MPa)	(mm)	(MPa)	(MPa)	(MPa)	(MPa)
SBRPMC 1%-35	3	Average	10.38	0.05	12.24	0.696	11.96	10.53	9.12	7.74
		STDEV	0.05	0.00	0.96	0.31	0.95	1.10	1.16	1.81
SBRPMC 1.5%-35	5	Average	9.53	0.05	15.22	0.671	14.61	13.53	11.86	10.52
		STDEV	0.85	0.00	1.49	0.33	1.22	1.71	1.63	1.48
SBRPMC 2.0%-35	3	Average	9.80	0.05	17.05	0.789	16.47	15.99	14.57	12.78
		STDEV	1.25	0.00	1.38	0.09	1.20	1.21	1.02	0.79
PVAPMC 1.5%-35	3	Average	11.41	0.05	16.60	0.436	16.17	11.77	9.56	7.39
		STDEV	0.93	0.00	1.94	0.05	1.69	1.91	1.43	1.09
Conv. SBR PMC1.5%-35	3	Average	6.68	0.05	10.37	1.056	9.51	9.88	9.24	8.48
		STDEV	0.93	0.00	0.91	0.50	1.19	0.97	0.69	0.64
SBRPMC 1.5%-50	3	Average	9.72	0.05	16.76	1.194	15.29	15.91	14.7	13.23
		STDEV	0.97	0	1.87	0.301	1.96	1.68	1.25	1.21

Note: Conv. SBR stands for conventional, as opposed to roller compacted SBR concrete.
